# Supplementary material for: Angelica sinensis polysaccharide as potential protectants against recurrent spontaneous abortion: focus on autophagy regulation
Source: Front Med (Lausanne). 2025 Jan 15;12:1522503. doi: 10.3389/fmed.2025.1522503 (PMC11774876; doi:10.3389/fmed.2025.1522503)
Supplement: Supplementary material S1 — The main instruments used during the LC-MS process, along with their models/specifications and manufacturers. [file Supplementary_file_1.zip › Metabolomics sequencing data FC1.2/6.差异代谢物/heatmap/横聚类/Exposure_Ctrl.pdf]

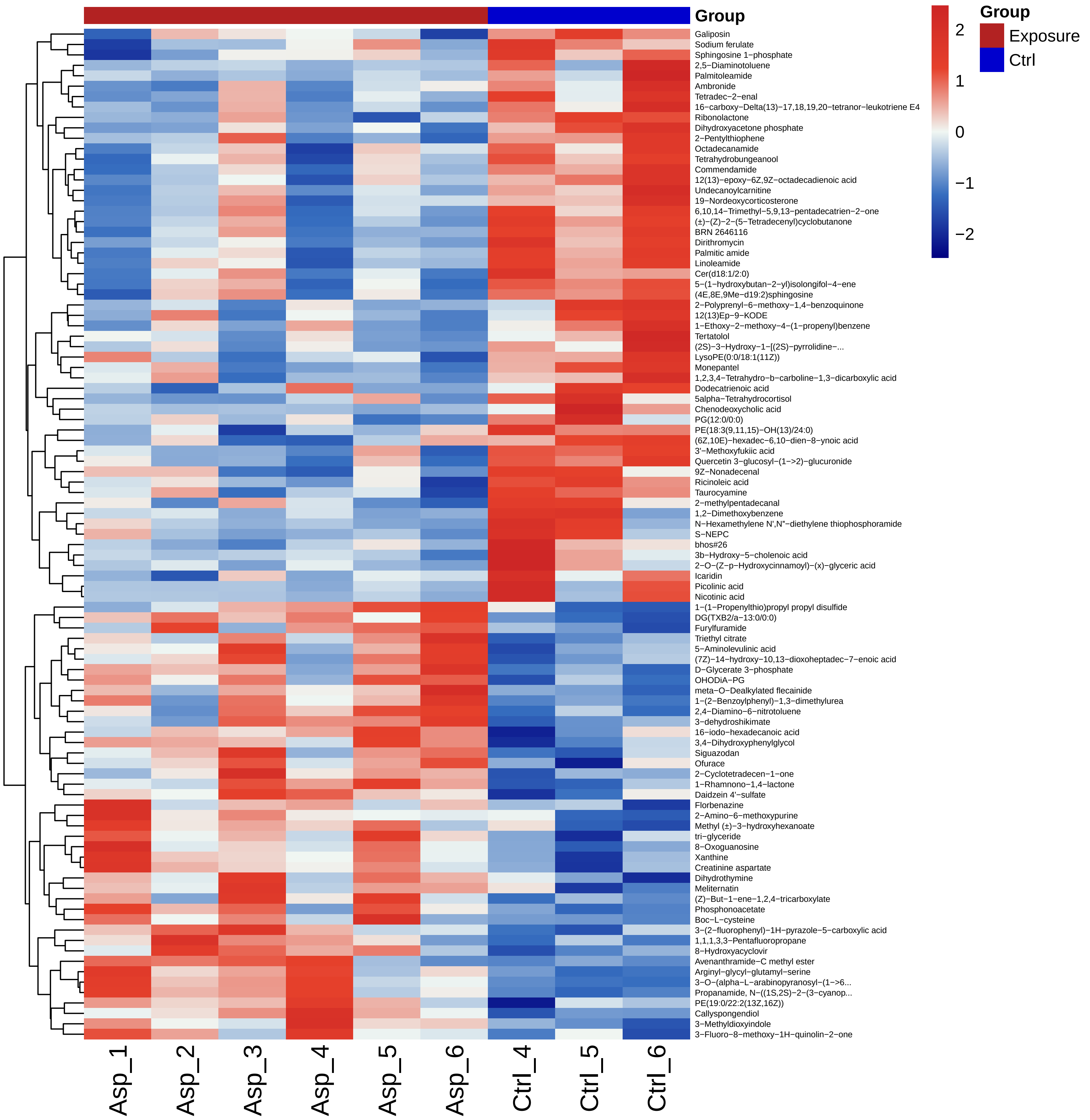

Group
Group
Exposure
Ctrl
2
1
0
−1
−2
Galiposin
Sodium ferulate
Sphingosine 1−phosphate
2,5−Diaminotoluene
Palmitoleamide
Ambronide
Tetradec−2−enal
16−carboxy−Delta(13)−17,18,19,20−tetranor−leukotriene E4
Ribonolactone
Dihydroxyacetone phosphate
2−Pentylthiophene
Octadecanamide
Tetrahydrobungeanool
Commendamide
12(13)−epoxy−6Z,9Z−octadecadienoic acid
Undecanoylcarnitine
19−Nordeoxycorticosterone
6,10,14−Trimethyl−5,9,13−pentadecatrien−2−one
(±)−(Z)−2−(5−Tetradecenyl)cyclobutanone
BRN 2646116
Dirithromycin
Palmitic amide
Linoleamide
Cer(d18:1/2:0)
5−(1−hydroxybutan−2−yl)isolongifol−4−ene
(4E,8E,9Me−d19:2)sphingosine
2−Polyprenyl−6−methoxy−1,4−benzoquinone
12(13)Ep−9−KODE
1−Ethoxy−2−methoxy−4−(1−propenyl)benzene
Tertatolol
(2S)−3−Hydroxy−1−[(2S)−pyrrolidine−...
LysoPE(0:0/18:1(11Z))
Monepantel
1,2,3,4−Tetrahydro−b−carboline−1,3−dicarboxylic acid
Dodecatrienoic acid
5alpha−Tetrahydrocortisol
Chenodeoxycholic acid
PG(12:0/0:0)
PE(18:3(9,11,15)−OH(13)/24:0)
(6Z,10E)−hexadec−6,10−dien−8−ynoic acid
3'−Methoxyfukiic acid
Quercetin 3−glucosyl−(1−>2)−glucuronide
9Z−Nonadecenal
Ricinoleic acid
Taurocyamine
2−methylpentadecanal
1,2−Dimethoxybenzene
N−Hexamethylene N',N''−diethylene thiophosphoramide
S−NEPC
bhos#26
3b−Hydroxy−5−cholenoic acid
2−O−(Z−p−Hydroxycinnamoyl)−(x)−glyceric acid
Icaridin
Picolinic acid
Nicotinic acid
1−(1−Propenylthio)propyl propyl disulfide
DG(TXB2/a−13:0/0:0)
Furylfuramide
Triethyl citrate
5−Aminolevulinic acid
(7Z)−14−hydroxy−10,13−dioxoheptadec−7−enoic acid
D−Glycerate 3−phosphate
OHODiA−PG
meta−O−Dealkylated flecainide
1−(2−Benzoylphenyl)−1,3−dimethylurea
2,4−Diamino−6−nitrotoluene
3−dehydroshikimate
16−iodo−hexadecanoic acid
3,4−Dihydroxyphenylglycol
Siguazodan
Ofurace
2−Cyclotetradecen−1−one
1−Rhamnono−1,4−lactone
Daidzein 4'−sulfate
Florbenazine
2−Amino−6−methoxypurine
Methyl (±)−3−hydroxyhexanoate
tri−glyceride
8−Oxoguanosine
Xanthine
Creatinine aspartate
Dihydrothymine
Meliternatin
(Z)−But−1−ene−1,2,4−tricarboxylate
Phosphonoacetate
Boc−L−cysteine
3−(2−fluorophenyl)−1H−pyrazole−5−carboxylic acid
1,1,1,3,3−Pentafluoropropane
8−Hydroxyacyclovir
Avenanthramide−C methyl ester
Arginyl−glycyl−glutamyl−serine
3−O−(alpha−L−arabinopyranosyl−(1−>6...
Propanamide, N−((1S,2S)−2−(3−cyanop...
PE(19:0/22:2(13Z,16Z))
Callyspongendiol
3−Methyldioxyindole
3−Fluoro−8−methoxy−1H−quinolin−2−one
Asp_1
Asp_2
Asp_3
Asp_4
Asp_5
Asp_6
Ctrl_4
Ctrl_5
Ctrl_6
